# Characterization of German sheep production systems and derivation of respective economic values for breeding traits

**DOI:** 10.1016/j.vas.2025.100432

**Published:** 2025-02-20

**Authors:** J. Oberpenning, K. Brügemann, S. König

**Affiliations:** Institute of Animal Breeding and Genetics, University of Giessen, Ludwig St. 21B, 35390 Giessen, Germany

**Keywords:** Sheep production systems, Cluster analysis, Breeding goals, Economic weights, Contingent valuation

## Abstract

The aim of this study was to derive economic weights for sheep breeding goal traits for specific cluster types. The herd clustering approach considered 25 German sheep herds including descriptors related to area-based factors, management factors, herd information and socio-economic characteristics. The evaluation criterion suggested the application of agglomerative hierarchical clustering with the respective allocation of 25 herds to 3 different clusters. The clusters mainly differed with regard to herd size, production focus, and organic versus conventional management practices. The ongoing contingent valuation approach to derive cluster specific economic weights for 12 breeding goal traits based on the willingness to pay (WTP) considering 25 German sheep farmers, a hypothetical budget of 1000 € for the improvement of a trait per 1 genetic SD, and linear mixed model applications. Significant cluster differences for the WTP (*P < 0.01)* were only identified for the trait feed utilisation with regard to clusters 2 (least-squares mean = 101.65 €) and 3 (least-squares mean = 9.46 €). Least-squares means for individual breeding goal traits were summed up into four overall breeding goal categories. The highest trait importance was expressed for “functional”, but displaying a variation among cluster. All clusters rated the trait category “health and welfare” including tail length very similar (range from 24 % to 29 %), with even stronger emphasize on “health and welfare” over “performance” in clusters 1 and 3. In consequence, we suggest to modify sheep breeding goals with a shift from production towards health, welfare and functionality, by consideration of production system particularities.

## Highlights

• Clustering approaches clearly separated the heterogeneous German sheep herds into 3 major production systems.
• The generated clusters (= production systems) can be used to improve genetic evaluations in sheep.
• The contingent valuation and willingness to pay approaches are suitable to derive economic weights for breeding goal traits reflecting health and welfare.
• It is imperative to implement cluster specific sheep breeding goals.

## 1 Introduction

Recently, Wiedemann et al. (2023) outlined the economic challenges of sheep production in Germany. In this regard, they highlighted the increasing importance of compensatory payments for agri-environmental service including landscape conservation. From a production perspective, most of the farm income is due to quality lamb meat production in niche markets (Mandolesi et al., 2020). In a comprehensive economic evaluation for the German ministry, von Korn (2020) indicated a profit of 55 € per ewe and year when considering the compensatory payments, but financial losses of 68 € per ewe and year without financial support.

Nevertheless, despite the economic challenges, sheep farming in Germany plays an important role in the German livestock sector. In total 1559,100 sheep from 35 different breeds (count in 2023) are kept in 9560 different farms (Schütte, 2023). A quite large number of 4310 small-sized farms keep less than 49 sheep, mostly in extensive or organic production systems. In contrast, only 850 large-scale herds with more than 500 sheep are listed in official statistics (Statistisches Bundesamt, 2023). Generally, especially with regard to production system characteristics and utilized breeds, sheep farming in Germany is very heterogeneous. Schütte (2023) highlighted nine different major husbandry systems for German sheep farming which were defined as follows: 1. “site-independent herding”, 2. “full-time site-bound herding farms”, 3. “full-time combined herding and paddock farms”, 4. “full-time sheep meat mixed farms”, 5. “full-time farms with focus on extensive meat production and landscape management”, 6. “part-time landscape managing farms”, 7. “part-time direct marketers”, 8. “small-scale breeders and self-suppliers, and 9. “small-scale lifestyle sheep farms”. The nine identified systems mainly differed with regard to herd size, the grazing area in hectares, the production focus (wool, meat or landscape conservation) and the overall breeding approach (pure breeding versus crossbreeding). The diversity of sheep farm types and sheep farming systems was outlined on the basis of within-country evaluations in Tunisia (Ibidhi et al., 2018), but also on a broader Mediterranean scale (Caballero, 2001).

Cluster analyses enables the identification and grouping of herds with similar production system characteristics, as applied for breeding objectives in cattle and pigs (Herold et al., 2021). The most relevant cluster approaches in the context of animal breeding include agglomerative hierarchical clustering (AHC), fuzzy clustering (FZC) and partition around medoids (PAM). A detailed description of the methodological aspects with regard to AHC, FZC and PAM applications is given by Herold et al. (2021). For all cluster approaches, the silhouette width is the mostly used standard evaluation criterion to assess the quality of clustering and to determine the optimal number of clusters. Gorgulu (2010) applied FZC clustering to classify dairy cow herds according to milk yield characteristics. Milán et al. (2003) focused on AHC and used economic data to group sheep herds in an international context. In an international breeding context, Weigel and Rekaya (1999) suggested so-called “borderless clustering”, i.e., basing breeding value estimations and breeding goal definitions on production system characteristics rather than on country borders.

Ideally and theoretically, economic weights for breeding goal traits should consider production system particularities and respective farm economy, as well as demands raised by consumers and the society (Amer, 1999; Conington et al., 2004). From a practical perspective, it is not feasible to derive economic weights for breeding goal traits for all specific farm types, but for overall production systems, as determined, e.g., via cluster analyses. So-called objective methods to derive economic weights require detailed information including revenues, costs, herd parameters and biological coefficients related to animal traits (Groen, 1989). However, for novel functional traits without market values (tail length, temperament or sheep behaviour), it might be challenging to set up appropriate profit functions. Especially organic farming aims on alternative functional breeding goal traits, encouraging the development of specific breeding indices. In this regard, Simianer et al. (2007) suggested to broadening existing organic breeding goals by including novel functional traits, but they also noticed the difficulties in deriving economic weights by applying objective approaches. An alternative method to derive economic weights for breeding goal traits is the so called “contingent valuation (CV) method”. The CV estimates reflect the respondent´s “willingness to pay” (WTP) using survey technique (Venkatachalam, 2004), taking into account non-market values of traits and products as well as optional values reflecting, e.g., the beauty or elegance of breeding animals for specific conformation traits (Schierenbeck et al., 2009). However, also in the context of breeding goal definitions based on CV and WTP, the expression “economic weight” have been applied widely for many species (e.g., Biermann et al., 2016). The main focus in such context is the WTP for a change per trait unit, e.g., with regard to alterations of productivity or the health status (Olesen et al., 1999). Modified CV methods have been applied to derive economic weights for pig and horse breeding goal traits (Bennett & Blaney, 2003; Edel & Dempfle, 2004; Rohr et al., 1999; Teegen et al., 2008).

Existing sheep breeding goals include performance traits (focus on daily gain, muscling score and back fat thickness), but ignore animal health and welfare indicators (Schäler et al., 2018). Legal regulations will affect future sheep breeding goal definitions, especially the need to breed sheep with short tails, i.e., to avoid tail docking of lambs. Connor and Cowan (2020) conducted a consumer survey addressing the topic of body part mutilations in pigs, calves, lambs, poultry and chicken. The consumer acceptance for such practice was quite low, stimulating the reformulation of breeding objectives and breeding goals. In New Zealand, Kongara et al. (2023) analysed data from a sheep farmer survey. In contrast to consumer opinions, tail docking and castration is considered as a common management practice. The practical breeders highlighted the importance of other breeding goal traits, indicating the need for a compromise, e.g., the development of sustainable breeding goals considering both productivity and novel welfare related traits.

In the context of the above-mentioned challenges, the objectives of this study were twofold. First, we focused on sheep herd-clustering approaches to allocate the herds to specific groups, which can be used for ongoing breeding objectives, e.g., production system specific genetic evaluations. Aiming on production system genetic evaluations, respective economic weights for breeding goal traits are imperative when setting up novel breeding indices. Hence, the second objective addressed the derivation of economic weights for sheep breeding goal traits, taking into account cluster particularities.

## 2 Material and methods

The present study incorporates two scientific parts. In a first part, cluster analyses was applied for the herd allocation into sheep production systems. In a second part, ongoing CV and WTP approaches were used to derive production system specific economic weights for breeding goal traits. The general database for both parts based on data recorded in 25 participating German sheep farms.

### 2.1 Herd and farm characterizations

The recorded major farm characteristics (MFC) used as input parameters for the cluster and WTP analyses are presented in Table 1. The MFC can be sub-grouped into A) area-based factors, B) management factors, C) herd information and D) socio-economic characteristics of the farmer. The area-based factors included the total cultivated land area in hectares, distinguished in meadows and pasture used for sheep grazing and the hectares used for crop production (arable land), as well as the number of farm sites. The farm management factors include the subsistence strategy (conventional versus organic), and the distinction in full-time or part-time farming. Further criteria to describe the farm management addressed the farm animal composition (only sheep or mixed farming with other species), the sheep husbandry system (herding without fencing, outside paddock husbandry or permanently keeping the sheep in the stable), and the number of farm employees. Herd information comprised the herd size, the production focus (meat production or landscape conservation), the number of lambings per year and the primary lambing season. Socio-economic characterizations focused on a direct description of the farmer including age and education in veterinary medicine or agriculture.

**Table 1:** Description of the major farm characteristics (MFC) from the 25 sheep farms (area-based factors, management factors, herd information and social -economic characteristics of the farmers).

Main category	Variable	Categories
A) Area-based factors	Farm sites	No. of farm sites
	Grassland	Yes No
	Arable land	Yes No
	Total area	ha
B) Management factors	Farming method	Conventional Organic
	Subsistence strategy	Full-time Part-time
	Other animals than sheep	Yes No
	Husbandry system	Stable husbandry Herding Paddock husbandry
	Employees	No. of employees
C) Herd information	Herd size	No. of sheep
	Production type	Meat production Landscape management
	Lambings per year	No. of lambings
	Lambing seasons	Spring Summer Autumn Winter
D) Social-economic characteristics of the farmer	Age of the farmer	Years
	Agricultural or veterinary education	Yes No

### 2.2 Herd clustering approaches

We applied and evaluated the following three herd clustering approaches AHC, PAM and FZC. All cluster analyses were conducted in R version 2023.09.1 (R Core Team, 2021), and applying the packages “cluster” (Maechler et al., 2023) and “ClustOfVar” (Chavent et al., 2022). In order to identify the best clustering method and to determine the optimal number of clusters, the overall evaluation criterion was the average silhouette width.

### 2.3 Contingent valuation method

#### 2.3.1 Survey to determine the willingness to pay for 12 breeding goal traits

The general structure of the questionnaire followed the approach as applied by Teegen et al. (2008) and Edel and Dempfle (2004) for horse breeding objectives, supplemented with criteria specific to sheep farming. The WTP for 12 breeding goal traits was assessed by identifying economic preferences of the 25 German sheep farmers. In this regard, each farmer had to allocate a fixed budget of 1000 Euro to the 12 possible breeding goal traits, following the question “which amount of money from the fixed 1000 Euro budget should be used to improve the respective breeding goal trait by 1 genetic standard deviation?” The genetic standard deviation per trait for the respective trait unit was communicated with the farmers, so that they experienced a practical impression for neutral trait comparisons. Overall, the breeding goal traits comprised the 4 categories production, functionality, health and welfare, and exterior traits. Performance traits included 1. Muscle and fat thickness (measured in cm, with a genetic standard deviation of 5.5 cm), 2. Daily weight gain (measured in g, with a genetic standard deviation of 265 g) and 3. Feed utilization (measured as feed conversion ration with a genetic standard deviation of 0.25). The functional trait category considered 4. Maternity (measured as weight gain in lambs (in kg) until day 42 with a genetic standard deviation of 3.5 kg), 5. Longevity (measured in ewes (in years) with a genetic standard deviation of 0.83 years), 6. female fertility (measured as litter size with a genetic standard deviation of 0.20) and 7. Landscape suitability (measured as daily basic activity for grazing and rumination (in hours) with a genetic standard deviation of 2.4 h). The category for health and welfare considered 8. Tail length (measured in cm with a genetic standard deviation of 2.05 cm) , 9. Claw health (measured as an incidence (in %) with a genetic standard deviation of 0.30 %) and 10. endoparasite resistance (measured in eggs per g faeces (a count) with a genetic standard deviation of 280 eggs). Exterior included 11. wool quality (measured as fibre diameter in cm with a genetic standard deviation of 0.5 cm) and 12. Conformation (subjectively scored in points with a genetic standard deviation of 0.4 points)

#### 2.3.2 Derivation of cluster specific economic weights for breeding goal traits

A linear mixed model applying the least square means procedure from the software package “lsmeans” (Lenth, 2016) was used to determine economic weights for breeding goal traits in the previously identified production systems (= clusters). All calculations were performed with R version 2023.09.1 (R Core Team, 2021).

The dependent variable *y* in this approach was the WTP per trait and respondent, implying 12 consecutive runs considering each breeding goal trait separately. The respective statistical model 1 was defined as follows:

(1) $$y_{ijklmn}=\mu +Breedtype_{i}+Numberofewes_{j}+Cluster_{k}+Farmerage_{l}+Farmereducation_{m}+e_{ijklmn}$$

where *y* *=* the WTP for the respective breeding goal trait; Breed type_i_= the fixed effect for meat- or land sheep (i= 1: meat production, i= 2 land sheep), Number of ewes_j_= the fixed effect for the number of ewes of considering 3 herd size classes (j= 1: < 100 ewes, j= 2: ≥ 100 and < 500 ewes, j= 3: ≥ 500 ewes), Cluster_k__=_ the fixed effect of the respective cluster of the respondent (k= 1, 2 or 3), Farmer age_l__=_ the fixed effect of the age class of the respondent (l= 1: ≤ 35 years, l= 2: 36–55 years, l= 3: ≥ 56 years), Farmer education_m_= the fixed effect for the agricultural or veterinary education of the respondent (m= 1: veterinarian/agriculture education, m= 2: no veterinarian/agriculture education), and *e_ijklmn_*= random residual effect.

In a final step, cluster-specific sheep breeding goals were constructed. In this regard, the lsmeans for the WTP per trait were summed up per cluster, and expressed in percentage in relation to the total WTP (= sum of all lsmeans for all traits within the respective cluster).

## 3 Results

### 3.1 Cluster approach comparisons

**Fig. 1:**
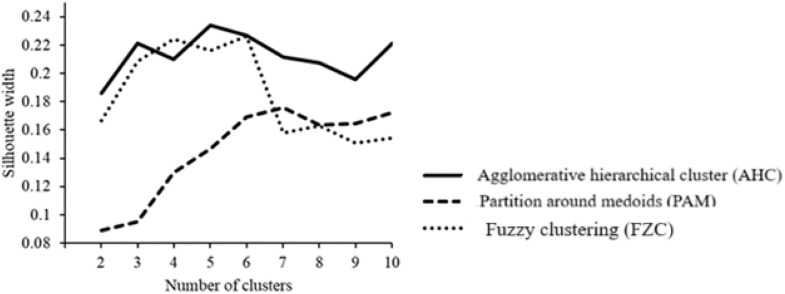
Average silhouette width for the three clustering methods agglomerative hierarchical cluster (AHC), partition around medoids (PAM) and fuzzy clustering (FZC) and the defined number of clusters.

**Fig. 2:**
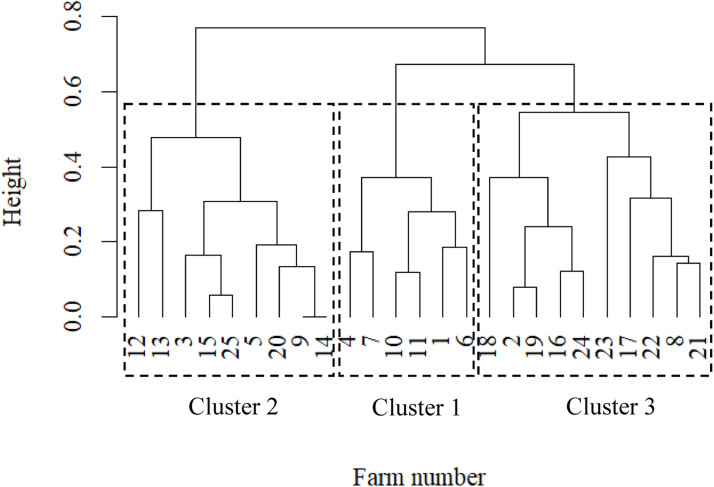
Dendrogram for the farm allocation via agglomerative hierarchical clustering.

With regard to the evaluation criterion “silhouette width”, the higher the value, the better the chosen method. Fig. 1 indicates the average silhouette width for the three methods AHC, PAM and FZC. Accordingly, the most suitable method indicating best fit to our data was AHC (black solid line). The first local maximum of the line indicates the optimal number of clusters, i.e., 3 clusters for AHC. Consequently, all ongoing analyses base on the results from the AHC method with 3 clusters. The dendrogram (Fig. 2) shows the allocation of the 25 sheep herds to the 3 clusters. The 25 farms are marked on the x-axis with their respective farm number, while the y-axis reflects the distances with regard to the silhouette width for farm comparisons. Six herds were allocated to cluster 1, 10 herds to cluster 2, and 9 herds to cluster 3.

### 3.2 Cluster description

A detailed description of the clusters with regard to the farm characterizations is given in Table 2. Cluster 1 includes the smallest number of farms (n= 6) and represents the medium-sized farms with only one farm site. The number of sheep kept in cluster 1 herds is very similar with cluster 3 (median of 103 ewes in cluster 1 herds (range from 50 to 200 ewes), and of 100 ewes in cluster 3 herds), but the total land area per farm comprising with a median of 280 ha is quite large in cluster 1. The cultivated agricultural area ranged from 45 ha to 912 ha. The majority of these farms focus on conventional management strategies, but with regard to between-cluster comparisons, cluster 1 includes the largest percentage of organic farms. All the farms from cluster 1 keep other animal species in addition to sheep, with predominant focus on one specific sheep breed. The majority of these farms use their sheep for meat production and keep them exclusively in stable- or paddock husbandry systems, with one lambing season per year. These farms allocated to cluster 1 have a medium number of employees (at average 5.3 persons) when compared to the cluster 3 farming system (at average 16.6 persons).

**Table 2:** Distribution of major farm characteristics in clusters 1, 2 and 3.

	Cluster 1	Cluster 2	Cluster 3
Number of farms	6	10	9
A) Area-based factors			
Farm sites			
Single	100 %	70.0 %	77.8 %
Multiple	0 %	30.0 %	22.2 %
Use of grassland	100 %	100 %	100 %
Use of arable land	83.3 %	60.0 %	22.2 %
Total area			
Median (ha)	280	189	20
Range (ha)	45 to 912	65 to 2380	10 to 88
B) Management factors			
Farming method			
Conventional	66.7 %	100 %	77.8 %
Organic	33.3 %	0 %	22.2 %
Subsistence strategy			
Full-time	100 %	100 %	0 %
Part-time	0 %	0 %	100 %
Other animals than sheep	100 %	70.0 %	11.1 %
Number of sheep breeds			
One sheep breed	66.7 %	30.0 %	44.4 %
Several sheep breeds	33.3 %	70.0 %	55.5 %
Husbandry system			
Stable husbandry	100 %	80.0 %	66.7 %
Herding	0 %	50.0 %	0 %
Paddock husbandry	100 %	80.0 %	100 %
No. of employees (average)	5.3	16.6	1.5
C) Herd information			
Number of sheep			
Median	103	525	100
Range	50 to 200	255 to 2500	56 to 350
Production focus on			
Meat production	66.7 %	50.0 %	33.3 %
Landscape management	33.3 %	50.0 %	66.7 %
Lambing seasons			
One lambing season	100 %	80.0 %	100 %
Several per year	0 %	20.0 %	0 %

The variables from category D) are not shown, because they were treated as extra effects in the model for the WTP and not included in the cluster analysis.

Cluster 2 includes the largest number of farms (n= 10), and all of them are full-time conventional farms. With regard to sheep breed diversity, 70 % of the farms allocated to cluster 2 keep more than one sheep breed. A further characteristic of a quite large fraction of farms in cluster 2 is “mixed farming”, i.e., to utilize the sheep for meat production and for landscape conservation. The sheep are kept in stable- or paddock husbandry systems. The farm and herd sizes in cluster 2 are quite large, with at least 65 ha of land under cultivation and at average 525 ewes per herd (range: 255 to 2500 ewes per herd).

Cluster 3 exclusively includes the 9 part-time and hobby farms. The majority of these farms practice conventional farm management. The main production focus is landscape management, by completely ignoring herding. All of the herds practice a paddock husbandry system, keep at least 56 ewes, and cultivate maximal 88 ha of arable land.

### 3.3 Willingness to pay for breeding goal traits

The breed type which strongly reflects the production focus significantly affected (*P < 0.05*) the WTP for wool quality. The breeders focussing on meat production valued wool quality with 52.3 € ± 10.8 €, and the farmers keeping land sheep with 82.5 € ± 10.3 €. The lsmeans for the trait “suitability for landscaping” significantly differed (*P < 0.05*) between both breed types, i.e., with 64.1 € ± 17.5 € for the farms with focus on meat production, and with 117.4 € ± 18.2 € for the landscape maintenance production focus. The breed type (i.e., the production focus) also significantly affected the WTP for daily gain (*P < 0.05*). The respective lsmeans was 117.4 € ± € 18.2 € for the farms with focus on meat production, and 64.1 € ± 17.5 € for the landscape maintenance production focus. The age of the farmers significantly affected the WTP for endoparasite resistance (*P < 0.05*), with lsmeans of 84.7 € ± 23 € for the youngest age group, but 134.8 € ± 15 € for the older farmers. In this regard, also the education of the farmer significantly (*P < 0.05*) affected the WTP for endoparasite resistance. Interestingly, farmers without agricultural or veterinary education rated this trait significantly higher (124 € ± 13 €) than the farmer group with education (76 € ± 13 €). Hence, the older farmers without proved qualification in animal or veterinary science stronger emphasis breeding on endoparasite resistance than the younger farmers experiencing a modern education in agricultural or veterinary sciences. The other factors as included in model 1 were not significant (*P > 0.05*) with regard to WTP differences of the breeding goal traits. The number of ewes kept in the herds indicated very similar lsmeans for the respective breeding goal trait WTP, suggesting same breeding objectives for different herd size classes.

**Fig. 3:**
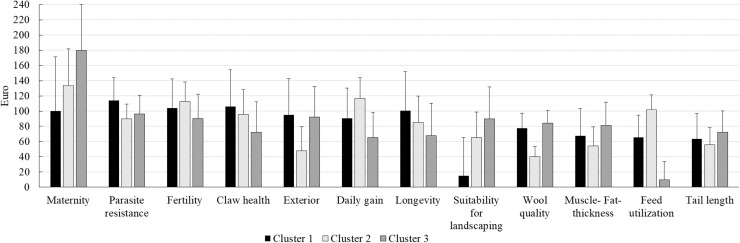
Least square means for the WTP of breeding goal traits in different clusters with respective standard errors.

The lsmeans for the WTP of the 12 breeding goal traits for the respective cluster are shown in Fig. 3. Among the 12 breeding goal traits, the cluster effect was only significant for the trait feed utilization (*P = 0.008*), with the highest WTP for feed efficiency in cluster 2. In cluster 1, the three breeding goal traits representing the highest values for WTP were endoparasite resistance with 114.2 € ± 17.6 €, claw health with 106 € ± 29.5 € and female fertility with 104.2 € ± € 22.8 €. The traits with lowest lsmeans for the WTP were landscape suitability (14.9 € ± 30.2 €), tail length (63.5 € ± 19.7 €) and feed utilization (65.39 € ± 19 €). The most important breeding goal traits in cluster 2 included maternity (134 € ± € 40.3), daily gain (116.8 € ± € 21.8 €) and female fertility (112.8 € ± 20.1 €). In contrast, of minor importance were wool quality (40.4 € ± 12.9 €), exterior (47.7 € ± 24.9 €) and muscle- fat thickness (54.4 € ± 19.4 €). With regard to cluster 3, the breeding goal traits reflecting highest lsmeans for the WTP included maternity (180 € ± 43.1 €), endoparasite resistance (96.1 € ± 16.6 €) and female fertility (90.5 € ± 21.5 €). The three traits with minor breeding goal importance in cluster 3 were feed utilization (9.46 € ± 17.9 €), daily gain (65.1 € ± 23.3 €) and longevity (67.5 € ± 30.2 €).

**Fig. 4:**
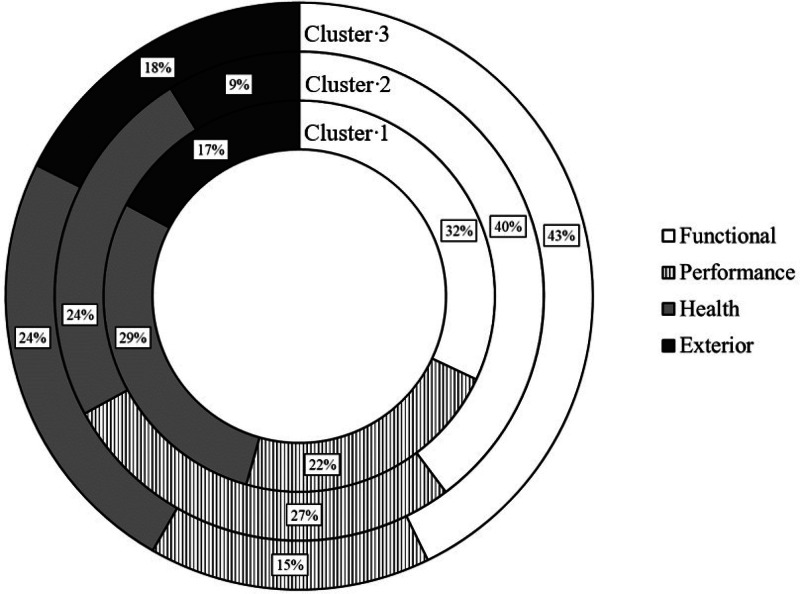
Relative weighs for the overall trait categories functionality, performance, health and welfare, and exterior in breeding goals of the three different clusters.

The relative emphasize for the WTP of the breeding goal traits per cluster is expressed in terms of the four overall categories production, functionality, health and welfare, and exterior in Fig. 4. In this regard, the general highest trait importance was expressed for “functional”, but displaying a variation across the three cluster (32 % economic weighting in cluster 1, 40 % economic weighting in cluster 2, 43 % economic weighting in cluster 3). All clusters rated the trait category “health and welfare” including the novel traits such as tail length very similar (range from 24 % to 29 %), with even stronger emphasize on “health and welfare” over “performance” in clusters 1 and 3. The classical “exterior” category including wool quality and conformation was of minor importance in cluster 2 (9 %). Accordingly, in clusters 1 and 3, the relative weights of exterior in the overall breeding goal were lower compared to “functional” and “health and welfare”. “Performance” displayed a moderate importance in the breeding goal of cluster 2 (27 %), but a relative economic weight of only 15 % in cluster 3.

## 4 Discussion

This study combines characterizations of sheep farms with a cluster analysis, contributing to a deeper understanding of sheep production particularities in Germany. The ongoing WTP approach for breeding goal traits based a contingent survey technique, aiming on the implementation of production system (= cluster) specific breeding strategies. The respective implications are discussed and contextualized in the following sections.

### 4.1 Cluster approach comparisons

Most of the clustering approaches addressing animal science topics focused one specific method, i.e., either AHC, PAM or FZC (Gorgulu, 2010; Köbrich et al, 2003; Schütte, 2023). The optimal cluster number generally depends on three major influential factors, i.e., a) the applied cluster method, b) the number of herd characteristics, and c) the number of farms considered in the study (Herold et al., 2021). In the present study, one focus was the evaluation of the different cluster approaches with regard to sheep production system classifications. The overall evaluation criterion was the average silhouette width, allowing the optimal consideration and combination of different farm characteristics with different measurements units such as count data, yield measurements or pure herd descriptions. Fig. 1 shows the comparison of the clustering methods based on the silhouette width, indicating superiority of AHC. Consequently, as suggested by Kaufmann and Rousseeuw (1990) for similar number of clustering elements (i.e., sheep herds in the present study), we chose the clusters generated via AHC for the ongoing breeding approaches. Although PAM and FZC enable the simultaneous consideration of different input data types (e.g., binary data, Gaussian distributed data, count data, etc.), the number of farms is the most relevant parameter contributing to the average silhouette width (Maione et al., 2019). Herd allocations to the different clusters indicated some differences, depending on the applied clustering approach FZC, PAM or AHC. Especially when comparing herd allocations from FZC and PAM, cluster compositions differed. With the AHC application, a clear distinction between the three clusters was possible, indicated through the obvious vertical distances in the dendrogram (Fig. 2). Distance calculations in AHC base on the “gower”-distance (Gower, 1971) as modified by Struyf et al. (1997), optimally taking into account the different scale levels and measurement units (Pavoine et al., 2009). Consequently, the AHC superiority in the present study might be due to the heterogeneity of input parameters (area-based factors plus management factors plus herd information plus social characteristics). To our knowledge, FZC was applied in other species with focus on only one or two types of input parameters, but not in sheep. Gorgulu (2010) used FZC to allocate 136 dairy cows into 4 clusters according to 6 cow characteristics including only “individual cow variables”, i.e., lactation number, 305-day milk yield, age at first insemination, age at first calving, the length of the dry period and calving interval. In our study, the average silhouette width for FZC was smaller compared to the average silhouette from the AHC application. A possible explanation addresses the chosen herd characteristics. Gorgulu (2010) solely focused on individual cow variables, by completely ignoring environmental or average herd characteristics. For such objective, FZC might be the most suitable approach (Herold et al., 2021).

Schütte (2023) applied the PAM herd clustering approach, and allocated 359 German sheep herds to 9 different clusters reflecting distinct production system characteristics. In contrast to our approach with only 2 or maximal 3 possible options per question, the survey by Schütte (2023) implied a broader range of possible answers per characterization parameter. For example, with regard to the input parameter “utilization”, we considered the options “meat production” and “suitability for landscaping”, but Schütte (2023) additionally included herds with focus on “wool production” and on “milk production”. However, a larger number of herds included in the survey enables a larger variety of possible answers per questions, ultimately contributing to the preference of a specific clustering approach.

In Tunisia and on the basis of 1025 sheep farms, Ibidhi et al. (2018) identified 5 sheep production systems and 4 distinct feeding systems using principal components analysis (PCA) and multivariate *k*-means classification. The identified cluster characteristics differed from the results in our study, because most of the considered herd input parameters were related to feed supply, feed resources and feed availability. Milán et al. (2003) used a 2-step approach considering PCA for a first categorization of farm types, and afterwards, on the basis of an overall categorisation, they applied AHC clustering. Input data from 52 sheep farms in Spain considered characteristics being related to our MFC: total surface (ha), arable land (ha), production focus, herd size and age of the farmer. In their clustering approach, 4 cluster groups were formed on the basis of 41 variables. Toro-Mujica et al. (2015) characterized sheep production systems in Chile by applying AHC. In analogy with our AHC results, 3 major production systems (cluster) were identified, mainly differing with regard to herd size, sheep / cattle ratio and agricultural particularities. Similar to our study, 14 variables were used for the analysis.

The four main farm characteristics used for sheep farm clustering in the present study were also considered by Riveiro et al. (2013) to categorize 44 Spanish dairy sheep herds, but the number of questions was much larger (in total 190 herd variables). Riveiro et al. (2013) defined 6 distinct clusters: large traditional farms, small traditional farms, farms with complementary agricultural activities, emerging small farms, modernized medium-sized farms and dairy sheep farms without arable land. Osorio-Avalos et al. (2015) used the Ward clustering approach (Ward, 1963) to allocate Merino sheep herds with similar environmental conditions into distinct groups to improve the reliability of genetic evaluations through the consideration of larger contemporary groups. For clustering, they considered 4 climatic variables, 10 variables related to managing systems and 6 variables reflecting the productivity of the herd. In analogy to our study, Osorio-Avalos et al. (2015) considered 25 sheep herds. However, in contrast to the quite homogeneous herds included in their dataset, the heterogeneity of herd characteristics contributed to a clearer separation of clusters in our present study. An alternative for simultaneous consideration of classical environmental and animal characteristics might be the application of a 2-step cluster analysis. In this regard, for the classification of 2024 organic dairy cattle herds, Ivemeyer et al. (2018) considered topographic and structural herd characteristics in a first step, and afterwards in step 2, the cow-related factors such as productivity.

### 4.2 Interpretation of cluster characteristics

The cluster analysis clearly indicated 3 production systems for sheep husbandry in Germany, with a main separation criterion according to herd size. Cluster 1 is comprised of medium-sized family farms, cluster 2 of large-scale farms, and cluster 3 of small farms with focus on sheep husbandry from a leisure time perspective. Nevertheless, a substantially larger percentage of farms is represented in clusters 2 and 3, supporting the classification by Schütte (2023), i.e., a fraction of 65 % of small and medium sized herds versus 35 % large-scale sheep herds in Germany. In all clusters, utilization of grasslands for the sheep played a dominant role, but differences in the utilization of arable land are obvious. Arable land as an additional source for forage production comprised more than 60 % of all herds in clusters 1 and 3, but only 22.3 % in cluster 2. Grazing of sheep on pasture still is a common farming practice in Germany as outlined in a comprehensive survey almost 20 years ago (Statistisches Bundesamt, 2010). Cluster particularities are due to the utilization of arable land in a feeding context. Especially the small and medium-sized herds as allocated to clusters 1 and 3 are seeking for alternative food resources due to the scarcity of areas for pasturing. The different clusters generally focus on different management practices. The large-scale herds from cluster 2 with access to large grazing areas have an exclusive focus on conventional farming practices, while the percentage of organic farming is increasing in the small and medium-sized sheep herds in Germany. In cluster 3 (representing the type of “hobby farming”), organic agriculture is accompanied with part-time sheep farming as an additional source to increase farm income. The herds allocated to clusters 1 and 2 are exclusively managed in full-time. Furthermore, there are notable differences with regard to the distribution of housing systems across the three clusters. Herding is a common practice exclusively used in large-scale herds (cluster 2), which are typically suited to accommodate large flocks. Accordingly, the herd management characteristics were emphasized by Schütte (2023) with regard to sheep herd classifications. Herd management differences were intensively addressed for sheep herd characterization in Spain (Riveiro et al., 2013), but with focus on other factors including the type of buildings and facilities, status of mechanization and digitalization. In consequence, due to the country specific particularities describing sheep farming, there are limitations for a borderless clustering approach in an international context as suggested for dairy cattle (Jaeger et al., 2019). In Germany, the obvious distinction between full-time (herds in clusters 1 and 2) and hobby sheep farming explains the different production focus, i.e., the priority in landscape management (cluster 3) versus meat production (clusters 1 and 2). Nevertheless, there is an increasing trend in Germany to utilize sheep farming for landscape conservation, especially in regions with declining cattle populations and poor yields from crop production, indicating the growing importance of adaption and female fertility instead of productivity (Gernand et al., 2008).

### 4.3 Willingness to pay for breeding goal traits

The significant cluster effect differences for the WTP for feed utilization between cluster 2 (101.65 € ± 16.7 €) and cluster 3 (9.46 € ± 17.9 €) supports the above mentioned breeding goal particularities with regard to the production focus. Cluster 2 only includes full-time farms with a strong focus on profit maximization through lamb meat production, explaining the strong importance of feed utilization. In such context, i.e., increasing the income through improved meat production, Barros de Freitas et al. (2021) suggested the new breeding traits residual water intake and residual feed intake in addition to feed utilization. A further classical production trait is daily gain. Consequently, the WTP for daily gain was larger in cluster 2 than in cluster 3, because daily gain plays an inferior role for landscape conservation. The WTP for the production trait muscle and fat thickness was on a similar level across clusters. Clelland et al. (2014) suggested to focus on sheep meat quality (especially on intramuscular fat content) instead of pure muscle content, to accelerate income from meat production on specific niche markets. The importance of meat quality traits when producing for nice markers was highlighted for local pig breeds in the WTP study by Biermann et al. (2016). Among all the traits considered in the present sheep WTP approach, production traits displayed the largest heritabilities (Justinksi et al., 2023). Hence, a large economic weight for large heritability traits implies large genetic gain, and vice versa, only small or moderate WTP for low heritability functional traits only marginal selection response, especially in the case of genetic antagonistic trait relationships (König et al., 2013).

The WTP for tail length as a novel breeding goal trait from the category “health and welfare” was of moderate and similar importance in all clusters (55.9 € to 72.2 €). The necessity for breeding on short tails due to direct and indirect effects on sheep wellbeing was highlighted in several recent studies (e.g., Johnson et al., 2023). From an animal health and welfare perspective, clear causalities in this regard were shown, i.e., detrimental effects of long and wooly traits on uterus infections, tail lesions and fractures, and further injuries. In the context of animal health and welfare, similar to tail length, the WTP for parasite resistance and claw disorders were in a range from 89.7 € to 114.2 €, displaying non-significant differences across clusters. Interestingly, we found a significant effect of the farmers’ age and education on the WTP for the trait parasite resistance. One explanation for the stronger breeding goal importance of parasite resistance in the older farmer age class might be related to the practical experiences by the shepherds and their motivations for the continuous improvement of pasture management strategies without utilization of anthelmintics (Häring et al., 2008). In an international context, e.g. in Uruguay, parasite resistance is already included in overall breeding objectives (Ciappesoni et al., 2023). In addition to endoparasite resistance, phenotypic and genetic selection on improved ecoparasite resistance, e.g., symptoms related to breech wrinkles, breech wool coverage and wool characteristics, have been suggested in the context of cutaneous myiasis (Bishop & Morris, 2007). However, the challenge to include a large number of novel functional traits into breeding objectives is related to the logistics, financial efforts and objectivity of phenotyping and trait recording (König & May, 2019). With regard to claw health in sheep, Lambertz et al. (2013) introduced new measurable characteristics, which were strongly related with footrot. Hence, there might be potential to modify existing breeding goals towards more health and animal welfare. Especially for the hobby farmers allocated to cluster 3, social aspects in the context of local and endangered breeds might have an economic value. For Washera sheep in West Gojam, Ethiopia, Hundie et al. (2022) applied a WTP approach in an across-breed perspective, and they identified that the WTP for conservation was related with the level of education and financial farm characteristics. Social-cultural aspects were also highlighted by Gizaw et al. (2009) for sheep breeding in an African context.

### 4.4 Setting up new breeding goals

The overall breeding value definition for sheep in Germany indicates a strong focus on performance traits (50 %) and conformation traits (35 %) (VIT, 2024). Nevertheless, according to our results for the WTP of the four overall trait categories, highest priority was assigned to the “classical” functional traits in the range from 32 % to 43 % across the three clusters. Also the novel functional traits including the trait category “health and welfare” indicate economic weightings similar to performance. Accordingly, Tabbaa et al. (2019) favored functional traits over morphological or exterior characteristics for local sheep breeds located in the Far East. Morphological body measurements or exterior traits only showed weak correlations with adaptive traits (Vanvanhossou et al., 2020), justifying their minor importance for the breeding goal definition in the present study. In sheep, in addition to morphological body traits, wool quality is considered as an exterior trait, but nowadays, wool quality only marginally contributes to the farm income (Krupová et al., 2014). However, some country or breed specific particularities are related with breeding trait preferences. For example in Greece, relative economic weights were larger for production than for functional traits (Theodoridis et al., (2018). In the present study, the complex of novel health and welfare traits is of highest importance in cluster 3, comprising the “hobby farmers” with small sheep herds. Without focus on a specific product (meat), the health and welfare component mainly determines the farm profit for landscape conservation. Nevertheless, the category health and welfare played also a dominant role for meat sheep breeding in Ireland (Byrne et al., 2010), because of the indirect effect of health on productivity. In several studies, resistance to endoparasite or claw disease infections were associated with inefficiency of feed utilization, reduction in growth, and losses in daily gain or weights at different ages (Piedrafita et al., 2010). In agreement with the results from our study, the female fertility trait component (lambing difficulty, litter size) only had moderate importance for sheep breeding in Ireland (Byrne et al., 2010). As a novelty, our study is a first approach considering tail length in the health and welfare category, and aiming on the derivation of economic weights for tail length in sheep. In the context of the future legal aspects related to the routine of tail docking, breeding on short tails will be a major breeding focus, especially in European countries. Trait associations between tail length and other functional or production traits have been evaluated in several studies and sheep populations (e.g., Zhu et al., 2024; Teubes et al. 2023), but without addressing tail length in a breeding goals context. Nevertheless, in contrast to Germany, some other countries already defined overall sheep breeding goals and included an animal health and welfare component. For example in New Zealand, the overall breeding goal considers endoparasite resistance in addition to the “classical traits” lamb growth, meat yield, wooly quality and lamb survival rate till weaning (Sheep Improvement Limited, 2022). In Greece (Ragkos & Abas, 2015), health and welfare traits of the breeding index are the grazing ability and disease resistance. In Ireland, in agreement with the results from our study, the highest breeding goal weight is assigned to functionality, and sheep health is indirectly considered via the entries for medical treatments (Byrne et al., 2012).

## 5 Conclusion

The AHC clustering approach was superior in terms of the evaluation criterion “average silhouette width” compared to the PAM and FZC methods, and clearly allocated 25 German sheep herds into 3 distinct clusters. Main cluster differences were related to herd size, to the production focus (meat production or landscape conservation) and to organic or conventional herd management practices. Also social characteristics (education and age of the herd manager) played a role in this regard. Economic weights for breeding goal traits basing on the CV approach and the WTP, displayed the increasing importance of the trait categories functionality and health and welfare over classical production and exterior traits. Nevertheless, variations of economic weights for trait categories (e.g., a range from 32 % to 40 % for “functionality” across clusters) or of single trait importance (least-squares mean of 101.65€ for feed utilisation in cluster 2, but only 9.46 € in cluster 3) suggest specific breeding strategies for specific sheep production systems. Accordingly, genetic evaluations should be evaluated in ongoing studies in the context of possible genotype x production system interactions.

## Ethical statement

This breeding study is based on general farm information and costs and prices to derive economic weights. Hence, no specific animal care statement is required.

## CRediT authorship contribution statement

**J. Oberpenning:** Methodology, Investigation, Formal analysis. **K. Brügemann:** Software, Methodology, Investigation. **S. König:** Writing – review & editing, Funding acquisition, Data curation, Conceptualization.

## Declaration of competing interest

Herewith we declare that we have no conflicts of interest.
